# Certitude and controversy in the interdisciplinary treatment of acute angiocholitis

**DOI:** 10.1186/1471-2334-13-S1-P44

**Published:** 2013-12-16

**Authors:** Răzvan Vasile Stoian, Dan Nicolae Păduraru, Simona Elena Albu

**Affiliations:** 1 General Surgery Department and Emergency III, University Emergency Hospital of Bucharest, Romania; 2 Carol Davila University of Medicine and Pharmacy, Bucharest, Romania; 3 Obstetrics and Gynecology Department, University Emergency Hospital of Bucharest, Romania

## Background

Angiocholitis is, in almost all the cases, an acute bacterial infection of intrahepatic and extrahepatic biliary tract. In front of a patient with acute angiocholitis there are a multitude of therapeutic options, but until present it hasn’t been described an ideal therapeutic attitude because of the variety of aetiology, physiopathology and clinical manifestatios.

## Methods

This article presents the results of a four years prospective study (2008-2012) on patients diagnosed with acute angiocholitis in the emergency department of the University Emergency Hospital of Bucharest. The study was based on more than 20 years of clinical experience and collaboration between surgeons, gastroenterologists and infectionists.

## Results

We present the results of 87 patients with acute angiocholitis, evaluated and treated by a multidisciplinary team. The results are influenced mainly by the multiple therapeutic options and especially by how they were set for this pathology, for which there is no gold standard regarding the treatment.

The particularity of this study stems from the fact that although all patients were initially evaluated by a surgical team, the therapy was always established by a joint team surgeon - gastroenterologist – infectionist.

## Conclusion

Having a multidisciplinary team as the one we built in addressing these complex cases is prohibitive for most surgery clinics in county hospitals and even some health units in university hospitals, but the results are encouraging for the therapeutic sequence practiced in our clinic.

